# Fish-scale-derived carbon quantum dots coupled with ZnO/Co_3_O_4_ for adsorption-assisted visible-light decolorization of reactive black 5

**DOI:** 10.1039/d6ra05073e

**Published:** 2026-08-18

**Authors:** Javeria Asim, Muhammad Younas Afzal, Saeed Rehman, Ahson Jabbar Shaikh, Farooq Ahmad, Muhammad Tahir Amin, Fiaz Hussain, Muhammad Bilal

**Affiliations:** a Department of Environmental Sciences, COMSATS University Islamabad, Abbottabad Campus Abbottabad 22060 KPK Pakistan mbilal@cuiatd.edu.pk +92 333 5411536; b Department of Chemistry, COMSATS University Islamabad, Abbottabad Campus Abbottabad 22060 KPK Pakistan; c School of Environment and Resources, Laboratory of Solid Waste Treatment and Resource Recycle, Southwest University of Science and Technology Mianyang 621010 China; d Department of Chemical and Materials Engineering, College of Engineering, Northern Border University Arar 91431 Saudi Arabia; e Civil and Environmental Engineering Department, College of Engineering, King Faisal University Al-Ahsa 31982 Saudi Arabia mqdir@kfu.edu.sa; f Department of Mechanical Engineering, Gachon University Seongnam-si Gyeonggi-do 13120 South Korea fiaz415@gachon.ac.kr

## Abstract

Visible-light-responsive photocatalysts prepared from low-cost waste resources are attractive for the sustainable treatment of dye-contaminated wastewater. In this work, carbon quantum dots (CQDs) derived from fish scales were integrated with ZnO/Co_3_O_4_ to construct a ternary CQD-ZnO/Co_3_O_4_ nanocomposite for adsorption-assisted decolorization of reactive black 5 (RB5). ZnO and Co_3_O_4_ were prepared by wet-chemical precipitation and co-precipitation, respectively, while CQDs were obtained *via* hydrothermal carbonization of fish scales. The optimized ternary catalyst, denoted CZC (0.5CQDs-3ZnO/Co_3_O_4_), exhibited broadened visible-light absorption, an effective optical band gap of 2.62 eV, and markedly suppressed photoluminescence relative to the individual components, indicating improved interfacial charge separation. XRD, FTIR, SEM-EDX, TEM/SAED, and DLS analyses verified the coexistence of wurtzite ZnO, spinel Co_3_O_4_, and CQD-derived carbonaceous domains within a polycrystalline heterostructure, as well as their stable dispersion. Electrochemical impedance spectroscopy (EIS) revealed a lower charge-transfer resistance (*R*_ct_) in CZC, thereby facilitating efficient photocatalytic charge separation. RB5 removal depended strongly on pH, initial dye concentration, catalyst dose, and contact time. Fast dark adsorption contributed approximately 80% removal within 30 min, followed by visible-light-assisted decolorization to approximately 88% under optimized conditions; a maximum decolorization of 94% was obtained at pH 2. Adsorption followed pseudo-second-order kinetics and was better described by the Langmuir model, with an estimated maximum capacity of 72.5 mg g^−1^. Scavenger experiments indicated that hole (h^+^) and hydroxyl radical (^˙^OH) related pathways were important, and band-position analysis supported a CQD-mediated Z-scheme-like pathway. The catalyst retained 74% decolorization after three cycles. Exploratory machine-learning analysis further identified initial pollutant concentration as the dominant descriptor controlling removal efficiency. Overall, this study demonstrates that fish-scale-derived CQDs effectively enhance ZnO/Co_3_O_4_ performance, making the material a sustainable and promising candidate for industrial wastewater treatment.

## Abstract 2

Fish-scale biowaste-derived CQDs were coupled with ZnO/Co_3_O_4_. The optimized catalyst showed a 2.62 eV band gap, quenched PL, 94% decolorization at pH 2, and 74% activity after three reuse cycles.

## Graphical abstract

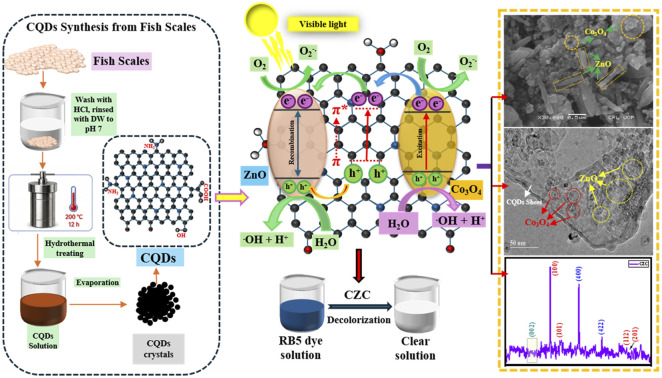

## Introduction

The discharge of synthetic dyes from textile, printing, leather, and related industries remains a persistent wastewater challenge because many dye molecules are highly colored, chemically stable, and resistant to conventional biological treatment.^1^ Azo dyes account for a major fraction of commercial textile dyes.^2^ Reactive black 5 (RB5), a representative anionic azo dye, contains aromatic rings and azo linkages that make it difficult to remove or transform under mild conditions.^3,4^ Even at low concentrations, RB5 can reduce light penetration in water bodies, disrupt aquatic ecosystems, and raise concerns about toxicity and human exposure.^5^ Effective and economically feasible treatment strategies are therefore required before dye-containing effluents are discharged.

Conventional dye-removal technologies include adsorption, membrane filtration, coagulation, and photodegradation-based processes.^6–9^ Adsorption is attractive because it is simple, rapid, and efficient for color removal; however, it often transfers pollutants from the liquid phase to a solid phase rather than fully degrading them.^10,11^ Advanced oxidation processes (AOPs) based on semiconductor photocatalysis can generate reactive oxygen species under light irradiation and may convert organic pollutants into less hazardous products. In practice, however, photocatalytic performance is limited by light-harvesting efficiency, recombination of photogenerated charge carriers, mass-transfer limitations, and the strong adsorption dependence of many dye molecules. For RB5, a combined adsorption-photocatalysis strategy is therefore more realistic than treating adsorption and photocatalysis as isolated processes. Various latest photocatalysts have been used in AOPs, including ZnO,^12^ CO_3_O_4_,^13^ ZnO/g-C_3_N_4_,^14^ CQDs/ZnO,^15^ ZrO_2_–ZnO,^16^ Biochar-ZnO,^17^ Cu/Zr Co-Doped Co_3_O_4_,^18^ ZnO@Mn_3_C,^19^ Sb_2_O_3_–CuO,^20^ for pollutant removal.

ZnO is widely investigated as a photocatalyst because of its low cost, abundance, high exciton binding energy, suitable valence-band oxidation ability, and chemical stability.^21^ Its wide band gap and rapid electron–hole recombination, however, restrict visible-light utilization and reduce photocatalytic efficiency.^22^ Coupling ZnO with a narrow-band-gap oxide is an effective route to extend the optical response and promote interfacial charge separation.^23^ Co_3_O_4_ is a p-type spinel oxide with visible-light absorption and redox-active Co^2+^/Co^3+^ centers, making it a useful counterpart for ZnO-based heterostructures.^24,25^ ZnO/Co_3_O_4_ composites can improve light harvesting and charge transport, but their performance may still be constrained by aggregation, limited charge mobility, and non-productive recombination at poorly controlled interfaces.^23,26^

Carbon quantum dots (CQDs) have emerged as versatile modifiers for semiconductor photocatalysts because they can broaden light absorption, mediate electron transfer, improve dispersion, and provide abundant surface functional groups.^27–29^ Biomass- and biowaste-derived CQDs are especially attractive because they integrate photocatalyst design with resource recovery.^30^ Fish scales are rich in carbon-containing biopolymers and inorganic residues, and hydrothermal treatment can convert this waste stream into CQDs containing sp^2^/sp^3^ carbon domains and oxygen- or nitrogen-containing groups. These functionalities may strengthen dye-catalyst interactions and create interfacial charge-transfer pathways when CQDs are coupled with oxide semiconductors.^31–33^

For ternary CQD-semiconductor systems, a mechanistic interpretation must be made carefully. CQDs may act as photosensitizers, electron reservoirs, conductive bridges, or adsorption-active interfacial layers depending on band alignment and surface chemistry.^34,35^ In this study, the charge-transfer mechanism is therefore proposed from UV-visible absorption, photoluminescence, band-position analysis, structural characterization, and scavenger experiments.

Herein, fish-scale-derived CQDs were integrated with a ZnO/Co_3_O_4_ heterostructure to form a ternary CQD-ZnO/Co_3_O_4_ nanocomposite for RB5 removal. The optimized 0.5CQDs-3ZnO/Co_3_O_4_ sample was evaluated using a combined structure–property–performance framework involving optical band-gap analysis, photoluminescence, XRD, FTIR, FESEM-EDX, TEM/SAED, DLS for confirming hydrodynamic size and zeta potential, adsorption kinetics and isotherms, operating-parameter optimization, scavenger testing, recyclability, and exploratory machine-learning-assisted descriptor ranking. The central objective was to clarify how CQD incorporation modifies ZnO/Co_3_O_4_ interfacial behavior and how adsorption and light-assisted photocatalysis jointly contribute to RB5 decolorization.

## Materials and methods

### Chemicals

All reagents were used as received without further purification. Distilled water was used throughout the synthesis, washing, adsorption, and photocatalytic experiments. The chemicals used in this study are summarized in Table 1.

**Table 1:** Chemicals used for photocatalyst synthesis and RB5 decolorization experiments

Reagent	Supplier	Specification
Zinc nitrate hexahydrate, Zn (NO_3_)_2_·6H_2_O	Daejung	≥98%
Sodium hydroxide, NaOH	Merck	≥95%
Sodium carbonate, Na_2_CO_3_	Merck	≥99%
Cobalt nitrate hexahydrate, Co (NO_3_)_2_·6H_2_O	Daejung	≥99%
Ethanol	Merck	≥99%
Hydrochloric acid, HCl	Merck	37 wt% aqueous solution
Reactive black 5 (RB5)	Across brand	Certified grade
Carbon black	Sigma-Aldrich	Conductive grade, ≥99%
PTFE dispersion	Sigma-Aldrich	60 wt% in H_2_O

### Synthesis of ZnO nanoparticles

ZnO nanoparticles were synthesized by wet chemical precipitation. Briefly, 0.2 M Zn(NO_3_)_2_·6H_2_O was dissolved in 400 mL of distilled water under continuous stirring for 1 h to obtain a homogeneous precursor solution. A 0.5 M NaOH solution was then added dropwise until the pH reached approximately 8, producing white Zn(OH)_2_ precipitates. The suspension was stirred for another 40 min to complete nucleation and growth. The precipitates were collected by centrifugation, washed three times with distilled water to remove residual ions, dried at 120 °C for 1 h, and calcined at 300 °C for 1 h to obtain ZnO nanoparticles (Fig. S1).

### Synthesis of Co_3_O_4_ nanoparticles

Co_3_O_4_ nanoparticles were synthesized by co-precipitation. Aqueous solutions of Co (NO_3_)_2_·6H_2_O and Na_2_CO_3_ were prepared separately and simultaneously introduced into a conical flask at 80 °C under continuous stirring at a 1 : 1 molar ratio. The precipitates were aged under the same thermal conditions for approximately 2 h and then cooled to room temperature. The solids were collected by centrifugation, washed, dispersed in ethanol, dried, and finally calcined at 300 °C for 2 h to improve crystallinity (Fig. S1).

### Synthesis of fish-scale-derived CQDs

CQDs were synthesized from fish scales through a one-step modified hydrothermal method.^84^ The fish scales were thoroughly washed, treated with diluted HCl to remove inorganic impurities, and then rinsed with distilled water until the wash solution reached a neutral pH. The cleaned scales were transferred to a Teflon-lined autoclave and hydrothermally treated at 200 °C for 12 h. During the hydrothermal process, the collagen-rich organic matrix of the fish scales underwent hydrolysis, dehydration, and carbonization, resulting in as-prepared fish-scale-derived carbon dots (CQDs).^36^ The resulting CQD-containing dispersion was centrifuged and filtered to remove coarse particulates and insoluble residues. The clarified dispersion, having a golden yellow color (Fig. S1), was then concentrated in a water bath at 60 °C to obtain the as-prepared fish-scale-derived golden yellow CQDs (Fig. S1). The recovered CQDs were used as obtained following centrifugation and filtration in all subsequent experiments. In the present batch, approximately 45 g of CQD product was obtained from 540 g of fish scales, corresponding to an 8.3% yield. The as-prepared CQDs were used directly for fabricating CQD-integrated ZnO/Co_3_O_4_ photocatalysts.

### Preparation of ZnO/Co_3_O_4_ and CQD-ZnO/Co_3_O_4_ nanocomposites

ZnO/Co_3_O_4_ binary nanocomposites were synthesized by a hydrothermal route using different ZnO-to-Co_3_O_4_ ratios (Table 2) (Fig. S2). For the 1 : 1 system, equal masses of ZnO and Co_3_O_4_ nanoparticles were dispersed in 100 mL of ethanol under continuous stirring. The suspension was transferred to a Teflon-lined autoclave and heated at 120 °C for 2 h. The resulting composite was washed with distilled water, centrifuged, and dried. The same procedure was used for the other ZnO/Co_3_O_4_ ratios.

**Table 2:** ZnO/Co_3_O_4_ binary nanocomposite systems were screened in this study

Sample code	Nominal ZnO ratio	Nominal CO_3_O_4_ ratio
1Zn : 1Co	1	1
1Zn : 2Co	1	2
1Zn : 3Co	1	3
2Zn : 1Co	2	1
3Zn : 1Co	3	1

**Fig. 1:**
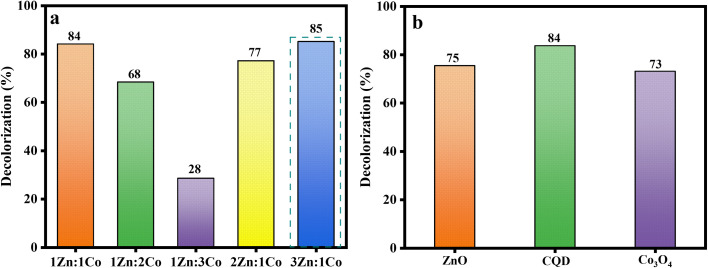
Screening of photocatalyst composition: (a) RB5 decolorization over different ZnO/Co_3_O_4_ ratios and (b) comparison of pristine ZnO, Co_3_O_4_, and CQDs.

The 3Zn : 1Co composition was selected for CQD decoration because it showed the highest initial decolorization performance among the tested binary composites (Fig. 1a). For CQD incorporation, 0.4 g of the 3ZnO/Co_3_O_4_ composite was dispersed in a 0.5 wt% CQD dispersion and stirred for 2 h (Fig. S3). The dispersion was sonicated for 40 min and then centrifuged for 20 min to remove the solvent. The recovered solid was dried at 100 °C for 10 h to obtain the ternary CQD-ZnO/Co_3_O_4_ nanocomposite. The optimized ternary material is denoted CZC throughout the manuscript.

### Photocatalyst screening

The ZnO/Co_3_O_4_ ratio had a significant impact on RB5 decolorization, according to screening studies of various compositions (Fig. 1a). Among various classifications, the 3Zn : 1Co binary compound removed 85% of the RB5 dye, whereas 1Zn : 3Co only removed 28%. This discrepancy suggests that too much Co_3_O_4_ might either increase recombination/aggregation or decrease the availability of powerfully oxidizing ZnO-derived holes (Fig. 12). Under the screening circumstances, CQD material outperformed compared to pure ZnO (75%) and Co_3_O_4_ (73%), providing 84–85% decolorization (Fig. 1b). The optical and PL results show that CQDs increase light harvesting and charge separation, which becomes significant under optimized operating circumstances and repeated usage, even though the absolute decolorization percentage improvement over the best binary composite is low. Different weights (%) ranging from 0–2% of CQDs were used with the ZnO–Co_3_O_4_ nanocomposite to evaluate the RB5 dye removal effectiveness. The ultimate optimized photocatalyst for RB5 decolorization was found to be 0.5%CQDs-3ZnO/Co_3_O_4_, which removed 86% of the RB5 in 60 minutes.

### Characterization

UV-visible absorption spectroscopy (Specord 200+) was used to evaluate optical absorption and estimate band-gap energies from Tauc plots. Photoluminescence (PL) spectroscopy (PerkinElmer LS-45) was used to assess radiative recombination behavior and emission features. Electrochemical Impedance Spectroscopy (EIS) (ZIVE POTENTIOSTAT/GALVANOSTAT/EIS) was used to evaluate solution resistance (*R*_s_) and charge transfer resistance (*R*_ct_). X-ray diffraction (XRD; Bruker D8 Advance) was used to identify crystalline phases and estimate average crystallite size from peak broadening. Fourier-transform infrared spectroscopy (FTIR; PerkinElmer) was used to identify surface functional groups and metal–oxygen vibrations. Field-emission scanning electron microscopy coupled with energy-dispersive X-ray spectroscopy (FESEM-EDX; JEOL JSM-6510LV) was used for morphology and elemental analysis. Transmission electron microscopy and selected-area electron diffraction (TEM/SAED; FEI Talos F200X) were used to examine nanoscale heterostructure formation and lattice fringes. Dynamic light scattering and zeta-potential measurements (Malvern Zetasizer) were used to evaluate hydrodynamic size and surface-charge behavior in aqueous dispersion.

### Electrochemical impedance spectroscopy (EIS)

Working electrodes were prepared by mixing 8.5 mg of active material (ZnO, Co_3_O_4_, or CZC) with 1 mg carbon black and 6.5 µL PTFE dispersed in 5 mL ethanol to form a homogeneous slurry. The slurry was coated onto a pre-weighed graphite sheet, dried at 60 °C overnight, and the active material loading was determined from the weight difference before and after coating. EIS measurements were conducted in a three-electrode cell using the prepared electrode as the working electrode, Ag/AgCl as the reference electrode, Pt wire as the counter electrode, and 0.1 M Na_2_SO_4_ as the electrolyte. The EIS measurements were carried out over a frequency range of 10 mHz to 100 kHz.

### Adsorption and photocatalytic decolorization experiments

Adsorption and photocatalytic experiments were performed using 50 mL of RB5 solution. Unless otherwise stated, the initial RB5 concentration was 30 mg L^−1^, the catalyst dose was 1 g L^−1^, and the initial pH was 7.32. Before irradiation, the suspension was magnetically stirred in the dark for 30 min to evaluate adsorption and approach adsorption–desorption equilibrium. Visible-light irradiation was then applied for the light-assisted decolorization stage. The RB5 concentration was monitored by UV-visible spectrophotometry using its characteristic absorption band. Decolorization efficiency was calculated using eqn (1), where *C*_0_ and *C*_*t*_ are the RB5 concentrations at time zero and time *t*, respectively.1
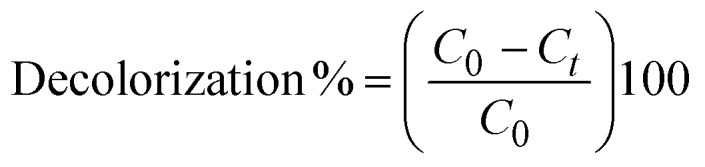


The adsorption capacity *q*_*t*_ at time *t* was calculated using eqn (2), where *V* is the solution volume and *m* is the catalyst mass.2
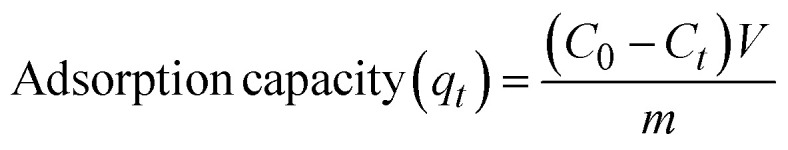


For optimization studies, the effects of photocatalyst composition, pH, initial dye concentration, catalyst dose, and contact time were examined. Scavenger tests were conducted using CaCl_2_, NaNO_3_, Na_2_SO_4_, and isopropanol (IPA) to probe the relative contribution of reactive pathways. Recyclability was evaluated over three repeated RB5 decolorization cycles.

### Adsorption and photocatalytic kinetics and isotherms

Adsorption kinetics were evaluated using pseudo-first order (PFO) and pseudo-second order (PSO) kinetic models,^37^ as described in eqn (3) and (4), respectively. Adsorption behavior was further interpreted using the Langmuir and the Freundlich isotherm models,^38^eqn (5) and (6). The photocatalytic decolorization kinetics of RB5 were assessed using an apparent pseudo-first-order expression, ln(*C*_0_/*C*_*t*_) = *kt* (eqn (7)).^39^ Because adsorption contributes substantially to the overall process, the kinetic constants are interpreted as apparent descriptors under the selected sequential dark-light conditions rather than intrinsic photocatalytic rate constants.

3 ln(*q*_e_ − *q*_t_) = ln *q*_e_ − *k*_1_*t*

4 *t*/*q*_t_ = 1/(*k*_2_ ln *q*_e_^2^) + *t*/*q*_e_

5
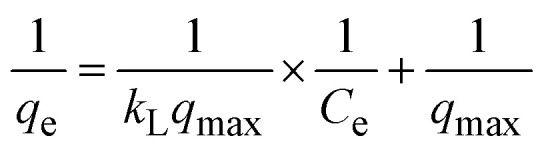
6
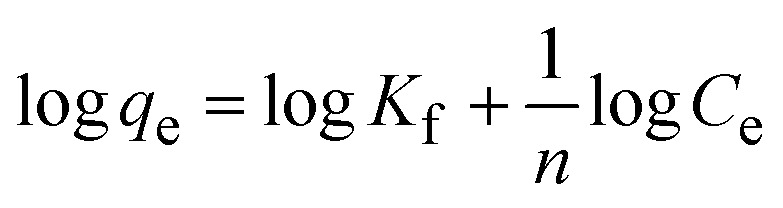
7
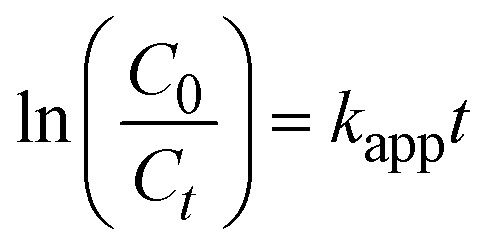
Here, *q*_e_ is the adsorption capacity at equilibrium (mg g^−1^), *q*_*t*_ is the adsorption capacity at time *t* (mg g^−1^), *k*_1_ and *k*_2_ are the PFO and PSO adsorption rate constants, respectively, *C*_e_ is the equilibrium adsorbate concentration in solution (mg L^−1^), *q*_max_ is the maximum adsorption capacity (mg g^−1^), *K*_L_ is the Langmuir constant related to adsorption affinity (L mg^−1^), *K*_f_ is the Freundlich constant related to adsorption capacity, *n* is the Freundlich intensity parameter, and *k*_app_ is the apparent rate constant for photocatalytic decolorization.

## Machine learning analysis

### Dataset preparation and descriptor definition

Machine learning techniques were employed to explore the experimental descriptors that affect the efficacy of CZC removal, using initial concentration of pollutants, solution pH, amount of photocatalyst added, and exposure time as input parameters. Pre-analysis data cleaning involved elimination of null columns, standardization of the column names, formatting of all the variables into numeric format, and omission of observations with missing targets (Fig. 14a). Features with uniform values across the dataset were also excluded because they do not add value in correlation or feature importance analysis.

### Permutation importance analysis

Permutation feature importance was additionally applied to evaluate the reliability of each descriptor ranking. In this method, one feature is randomly shuffled within the test set, and the resulting decline in model performance is measured. A larger decrease in *R*^2^ indicates that the shuffled feature contains more critical predictive information (Fig. 14b).

### Random forest feature-importance analysis

A Random Forest regression model was used to capture potential nonlinear descriptor effects that are not represented by Pearson correlation. The dataset was split into training and testing subsets, and the trained model was used to derive impurity-based feature importance, which measures the relative contribution of each descriptor to prediction performance (Fig. 14c).

### Correlation analysis

Pearson correlation analysis was used to evaluate linear relationships between the experimental descriptors and between each descriptor and removal efficiency. The Pearson correlation coefficient ranges from −1 to +1, with positive values indicating that two variables increase together, negative values indicating an inverse relationship, and values near zero indicating weak linear dependence. A correlation heatmap and individual target-correlation plot were generated to summarize these relationships (Fig. 14d).

## Results and discussion

### Optical absorption, band-gap response, charge-recombination behavior, and electrochemical impedance spectroscopy (EIS)

**Fig. 2:**
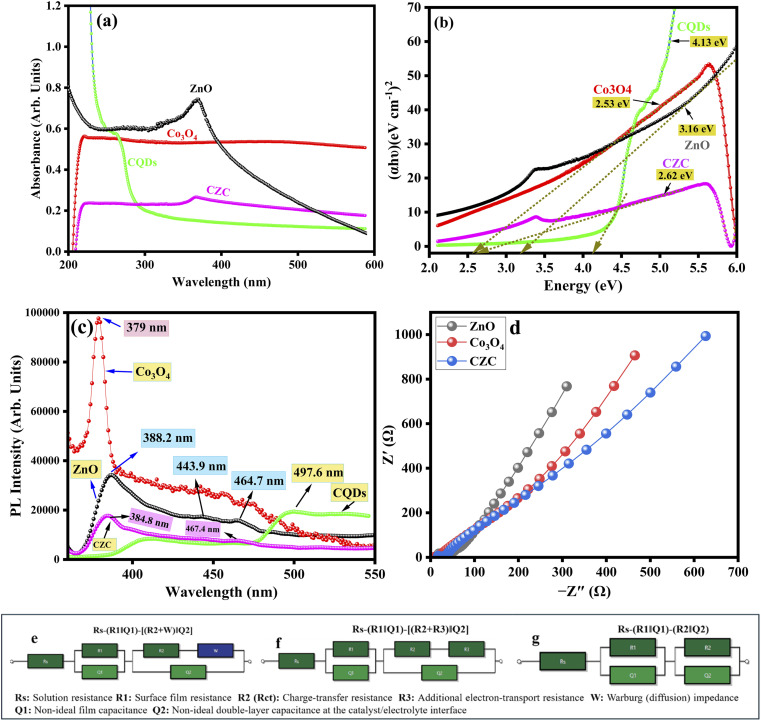
Optical properties of Co_3_O_4_, ZnO, CQDs, and CZC: (a) UV-visible absorption spectra, (b) Tauc plots and estimated direct band-gap energies, (c) photoluminescence spectra showing quenched emission from CZC; electrochemical analysis: (d) EIS measurements of ZnO, Co_3_O_4_, and CZC nanocomposite; equivalent circuits used for EIS data analysis (e) ZnO, (f) Co_3_O_4_, (g) CZC.

Fig. 2a compares the optical responses of ZnO, Co_3_O_4_, CQDs, and the ternary CZC nanocomposite. ZnO showed strong UV absorption consistent with its wide-band-gap semiconductor character (3.16 eV).^40^ Co_3_O_4_ exhibited stronger visible-light absorption owing to its narrower band structure,^41^ while CQDs showed intense short-wavelength absorption associated with π–π* and n–π* transitions of carbonaceous domains and surface functional groups.^42^ Compared with the pristine components, the synthesized CZC sample exhibited a broader and more continuous visible-light absorption response, which is compatible with the formation of the proposed CQD-ZnO/Co_3_O_4_ nanocomposite.^15,23^

Tauc analysis gave direct band-gap energies of approximately 3.16 eV for ZnO, 2.53 eV for Co_3_O_4_, 4.13 eV for CQDs, and 2.62 eV for CZC (Fig. 2b). The reduced effective band gap of CZC relative to ZnO supports improved visible-light activation after Co_3_O_4_ coupling and CQD decoration.

PL spectra further support improved charge separation.^43^ Co_3_O_4_, ZnO, and CQDs showed distinct emission features at approximately 379, 388.2, and 497.6 nm, respectively (Fig. 2c). In contrast, CZC displayed much weaker emission bands at 384.8 and 467.4 nm. The quenched PL intensity of CZC indicates suppressed electron–hole recombination and enhanced interfacial charge transfer (Fig. 2c), providing evidence for successful nanocomposite formation.^44^ The slight blue shift of the near-band-edge PL emission from 388.2 nm (ZnO) to 384.8 nm (CZC), despite ZnO being the predominant component (3ZnO: 1Co_3_O_4_), suggests modification of the local electronic environment through interfacial interactions, consistent with nanocomposite formation (Fig. 2c).^45,46^ The main PL emission assignments are summarized in Table 3.

**Table 3:** PL emission assignments for ZnO, Co_3_O_4_, CQDs, and CZC

Peak emission	Emission region	Assigned transition or origin	References
379 nm (Co_3_O_4_)	UV	O 2p → Co 3d-related transition	47
388.2 nm (ZnO)	UV/NBE	Near-band-edge recombination	48
443.9 nm (ZnO)	Blue	CB → Zn-vacancy/defect-state transition	49
464.7 nm (ZnO)	Blue	Deep-level emission associated with defect states	50
497.6 nm (CQDs)	Green	Surface-state emission related to π–π*/n–π* states	51
384.8 nm (CZC)	UV	ZnO-related band-edge emission after coupling	15
467.4 nm (CZC)	Blue	Interfacial charge transfer and defect/surface states	26

Electrochemical impedance spectroscopy (EIS) was performed to investigate the interfacial charge-transfer behavior of the synthesized photocatalysts (Fig. 2d). The Nyquist plots exhibit depressed semicircles followed by inclined lines, indicating non-ideal capacitive behavior and diffusion-controlled processes. The high-frequency semicircle corresponds primarily to the charge-transfer resistance (*R*_ct_), whereas the inclined line in the low-frequency region is associated with Warburg diffusion arising from ion diffusion at the electrode/electrolyte interface.^52^

**Fig. 3:**
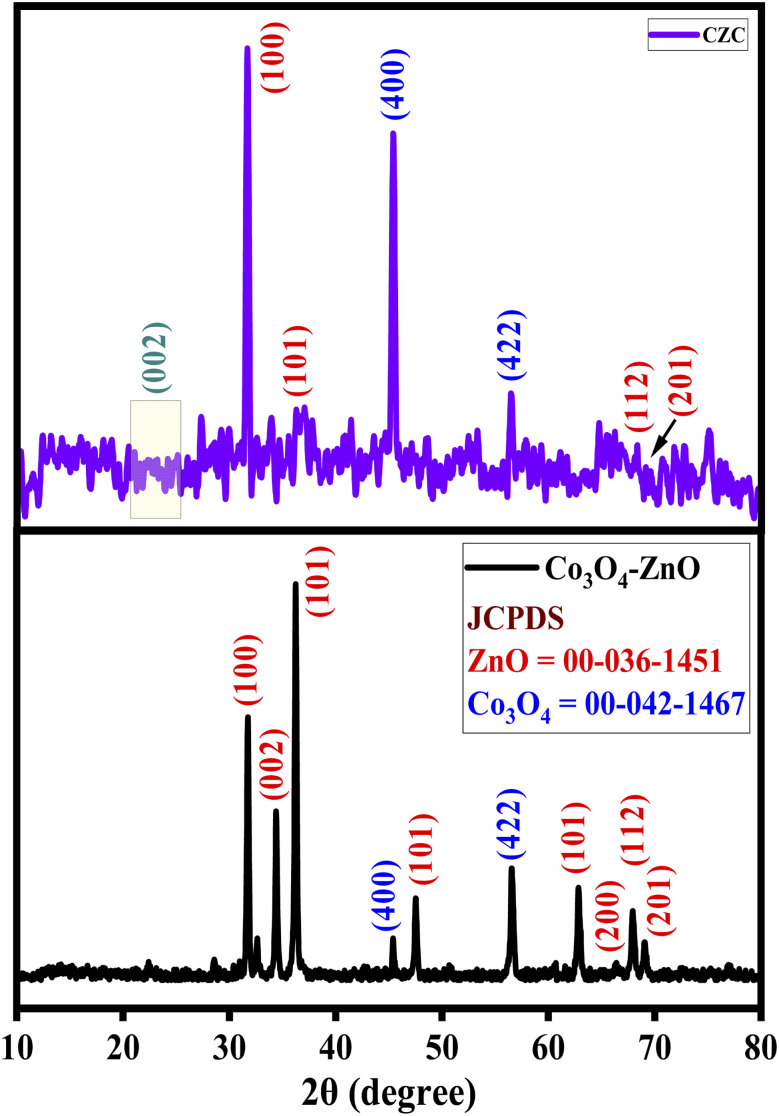
XRD patterns of ZnO/Co_3_O_4_ and CQD-decorated ZnO/Co_3_O_4_ (CZC) confirm the coexistence of wurtzite ZnO, spinel Co_3_O_4_, and CQD-related carbon domains.

**Fig. 4:**
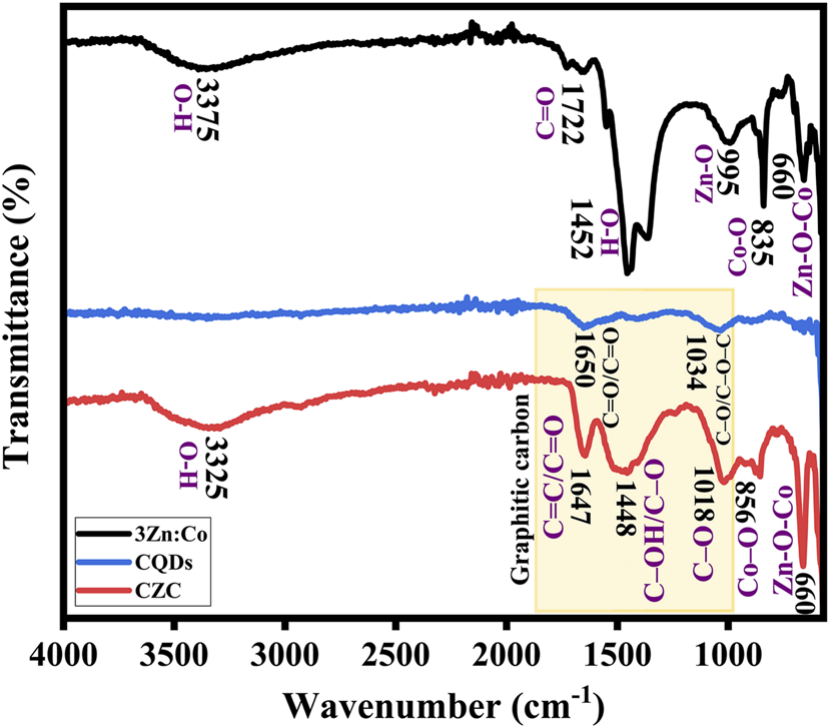
FTIR spectra of 3ZnO/Co_3_O_4_, CQD, and CZC, showing CQD-related functional groups and retained metal–oxygen vibrations.

The impedance spectrum was fitted using ZView software with equivalent circuits (Fig. 2e–g). ZnO was best fitted with the *R*_s_(*R*_1_‖*Q*_1_) − [(*R*_2_ + *W*)‖*Q*_2_] circuit, indicating that charge transfer was influenced by diffusion due to the presence of the Warburg element (*W*) (Fig. 2e). Co_3_O_4_ was described by *R*_s_ − (*R*_1_‖*Q*_1_) − [(*R*_2_ + *R*_3_)‖*Q*_2_], suggesting an additional resistive (*R*_3_) process associated with electron transport (Fig. 2f). In contrast, the CZC composite was adequately fitted using the simpler *R*_s_ − (*R*_1_‖*Q*_1_) − (*R*_2_‖*Q*_2_) circuit (Fig. 2g), indicating a more efficient interfacial charge-transfer process without requiring additional diffusion or resistance elements.^53^ The fitted charge-transfer resistance (*R*_ct_) values followed the order Co_3_O_4_ (37 Ω) > ZnO (32 Ω) > CZC (15 Ω), confirming that the incorporation of CQDs significantly facilitated interfacial electron transport and suppressed charge-carrier recombination.^54^ The lower *R*_ct_ of the CZC composite is consistent with its superior photocatalytic performance and is further supported by the reduced PL intensity (Fig. 2c), enhanced visible-light absorption in UV-visible (Fig. 2a and b), successful heterostructure formation confirmed by XRD (Fig. 3) and FTIR (Fig. 4), and the intimate interfacial contact observed in TEM (Fig. 6a and b), collectively demonstrating efficient charge separation and accelerated electron transfer. These results also validated the charge transfer mechanism (Fig. 12). The corresponding values of solution resistance (*R*_s_) and charge-transfer resistance (*R*_ct_) are mentioned in Table 4.

**Table 4:** Charge transfer resistance (*R*_ct_) and electrochemical resistance (*R*_s_) of different electrodes

Sample	*R*_s_ (Ω)	*R*_ct_ (Ω)
ZnO	222	32
Co_3_O_4_	227	37
CZC	222	15

### Crystal structure and phase composition

Fig. 3 shows the XRD patterns of the ZnO/Co_3_O_4_ binary composite and CZC. Diffraction peaks indexed to the (100), (002), (101), (102), (112), (200), and (201) planes confirm the hexagonal wurtzite phase of ZnO, while peaks associated with the (400) and (422) planes confirm the presence of spinel Co_3_O_4_.^55^ CZC retained the main oxide diffraction features, indicating that CQD decoration did not destroy the ZnO/Co_3_O_4_ crystalline framework. A broad CQD-related feature near 23° can be assigned to graphitic carbon domains corresponding to the (002) plane.^56^ The average crystallite size estimated from peak broadening decreased from 31.5 nm for 3ZnO/Co_3_O_4_ to 25.9 nm for CZC, suggesting that CQD incorporation introduced interfacial disorder and/or suppressed crystallite growth. The XRD-derived peak positions and crystallite sizes are summarized in Table 5.

**Table 5:** XRD-derived peak positions and crystallite sizes for 3ZnO/Co_3_O_4_ and CZC

Sample	Selected 2*θ* ranges	Average crystallite size (nm)
3ZnO/Co_3_O_4_	31.74, 34.41, 36.24, 45.41, 47.54, 56.59, 62.86, 66.37, 67.96, 69.07°	31.5 nm
CZC	31.66, 36.83, 45.38, 56.48, 66.37° and CQD-related broad feature near 23°	25.9 nm

### Surface chemistry and heterostructure morphology

FTIR analysis confirmed the presence of hydroxyl, carbonyl, graphitic carbon, C–O, Zn–O, and Co–O vibrational features (Fig. 4). The 3ZnO/Co_3_O_4_ spectrum showed lattice vibrations at approximately 995, 835, and 660 cm^−1^, attributable to Zn–O and Co–O bonds; bands between 1722 and 1452 cm^−1^ associated with C=O and O–H bending vibrations; and a broad band around 3375 cm^−1^ assigned to surface O–H stretching.^26,57^ The FTIR spectrum of CQDs showed characteristic peaks at 1653 and 1034 cm^−1^, corresponding to C=O/C=C and C–O functional groups, confirming successful CQD formation.^58^ The characteristic CQD bands shifted from 1653 and 1034 cm^−1^ to 1647 and 1018 cm^−1^ in the CZC composite (Fig. 4), accompanied by an O–H shift to 3325 cm^−1^, confirming successful CQD incorporation through strong interfacial interactions.^59^ The appearance of carbon-related bands confirms CQD integration, while the retained metal–oxygen vibrations indicate that the oxide framework was preserved.^45^

### SEM micrograph and EDX analysis of 3ZnO/Co_3_O_4_ nanocomposite and CZC

**Fig. 5:**
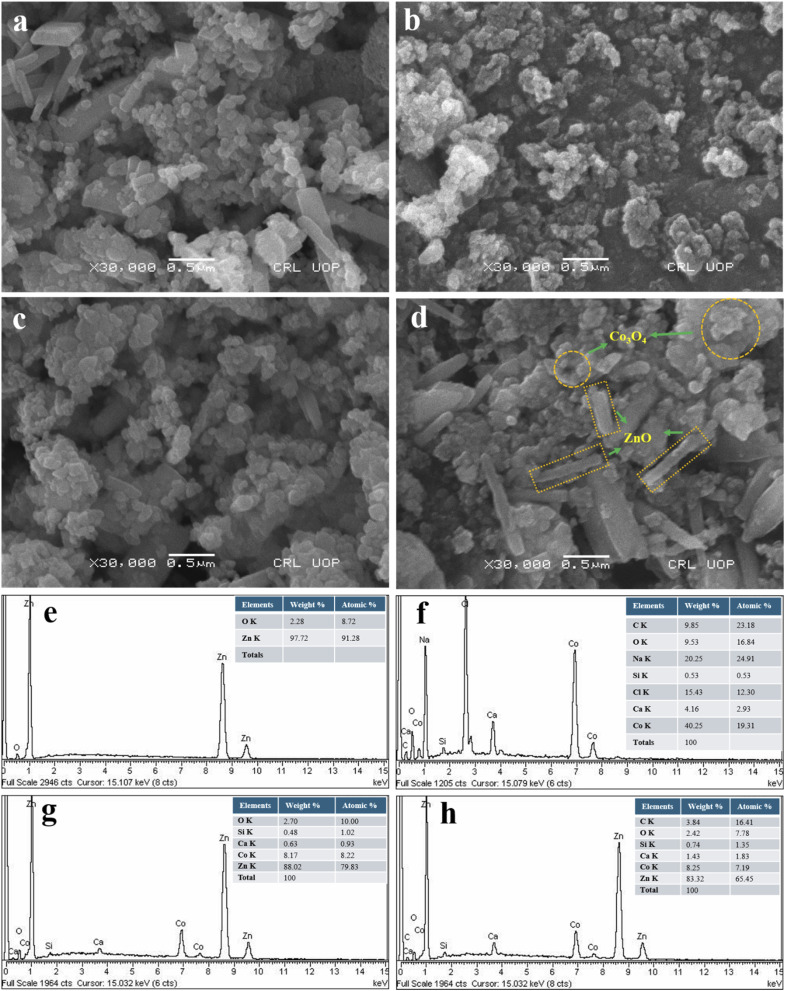
FESEM images: (a) ZnO, (b) Co_3_O_4_, (c) ZnO/Co_3_O_4_, (d) CZC, and EDX spectra: (e) ZnO, (f) Co_3_O_4_, (g) ZnO/Co_3_O_4_, (h) CZC nanocomposite.

**Fig. 6:**
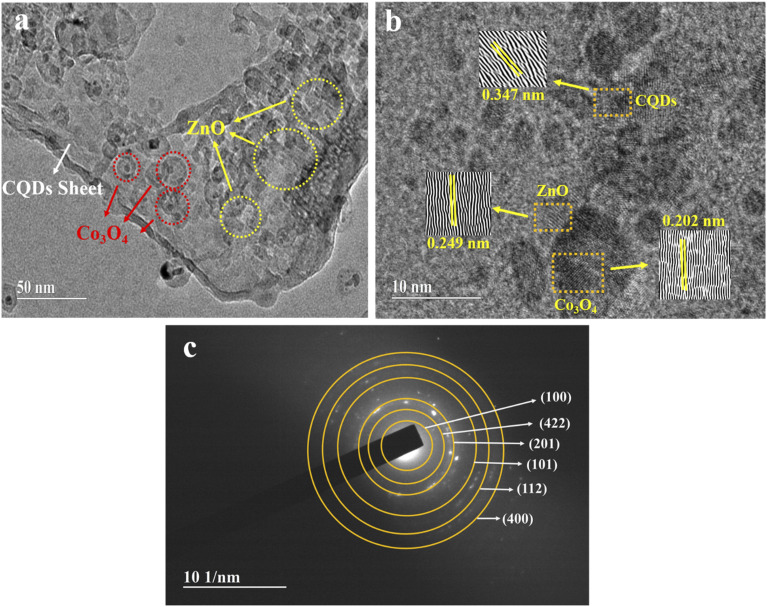
TEM analysis of CZC: (a) TEM image showing ZnO and Co_3_O_4_ anchored on CQD sheets, (b) TEM lattice fringes, and (c) SAED pattern confirming a polycrystalline heterostructure.

SEM-EDX characterization further supports heterostructure formation in CZC (Fig. 5). SEM images of pristine ZnO exhibit a predominantly hexagonal, rod-like morphology with slight agglomeration (Fig. 5a), while Co_3_O_4_ (Fig. 5b) exhibits a cauliflower-like morphology with pronounced agglomeration. The binary ZnO/Co_3_O_4_ composite exhibited hexagonal rod-like ZnO structures decorated with cauliflower-like Co_3_O_4_ aggregates (Fig. 5c). After CQD integration, ZnO rods and Co_3_O_4_ particles were embedded within a carbonaceous matrix attributed to CQDs (Fig. 5d), also confirmed from the TEM image (Fig. 6a). The EDX spectra of pristine ZnO and Co_3_O_4_ nanoparticles (Fig. 5e and f) confirm the presence of the expected elements, with only trace impurities detected in Co_3_O_4_, likely originating from the precursor materials. While CZC spectra confirmed the coexistence of Zn, Co, O, and C (Fig. 5h), the higher ZnO content (83.32 wt%) relative to Co_3_O_4_ (8.25 wt%) in the EDX analysis is consistent with the intended 3ZnO/Co_3_O_4_ (3 : 1) composition in the CZC. Minor Si and Ca signals were also detected, likely to originate from the fish-scale precursor used for CQD synthesis (Fig. 5d), and trace impurities from the precursor chemicals and substrate-related background contributions. Although EDX is semi-quantitative and surface-sensitive, the observed carbon signal is consistent with FTIR results and TEM observations, supporting successful incorporation of CQDs into the ZnO/Co_3_O_4_ system.

### TEM and SAED analysis of CZC

TEM/SAED data provide direct nanoscale evidence for the ternary CZC architecture. TEM images show ZnO and Co_3_O_4_ domains anchored on an aggregated CQD-derived carbonaceous network (Fig. 6a). Lattice spacings of approximately 0.347, 0.249, and 0.202 nm can be assigned to graphitic/CQD domains,^60^ associated with ZnO,^61^ and Co_3_O_4_,^62^ respectively (Fig. 6b). The SAED pattern contains multiple diffraction rings indexed to ZnO and Co_3_O_4_ planes, confirming a polycrystalline heterostructure^63,64^ (Fig. 6c). Such intimate contact is important because it shortens charge-transfer distances and allows CQDs to function as interfacial electron-mediating domains.

### Colloidal behavior of photocatalysts

**Fig. 7:**
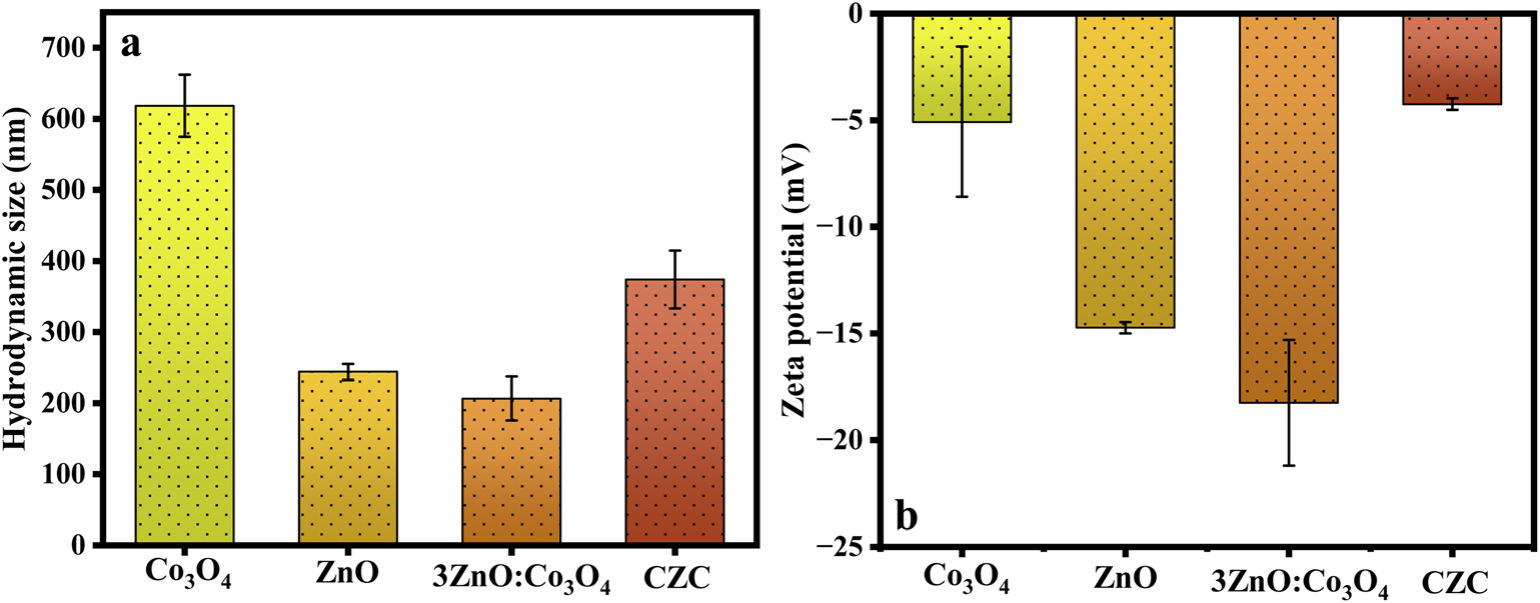
Colloidal properties of the synthesized materials: (a) hydrodynamic size distribution and (b) zeta potential.

The findings of dynamic light scattering are displayed in Fig. 7a and b. The order of the hydrodynamic size distribution was Co_3_O_4_ > CZC > ZnO > 3ZnO/Co_3_O_4_,^65^ suggesting that oxide coupling and CQD decorating changed the behaviour of colloidal aggregation (Fig. 7a). The larger hydrodynamic size of Co_3_O_4_ particles (618.5 ± 43.5 nm) can be supported by its lower surface charge (−5.07 ± 3.53 mV) (Fig. 7b), which promotes agglomeration (Fig. 7a). ZnO exhibited a zeta potential of −14.73 ± 0.26 mV and a hydrodynamic size of 243.7 ± 11.3 nm, indicating moderate colloidal stability. The 3ZnO/Co_3_O_4_ binary composite exhibited a more negative zeta potential (−18.3 ± 2.95 mV) and a smaller hydrodynamic size (206.4 ± 30.8 nm), suggesting improved electrostatic stabilization and reduced aggregation (Fig. 7b).^66^

Under the circumstances studied, all samples had negative zeta-potential values, indicating the presence of negatively charged surface groups (Fig. 7b). While the less negative surface of CZC (−4.23 ± 0.28 mV) and controlled hydrodynamic size (374.03 ± 40.8 nm) values may indicate CQD-derived surface groups and partial charge compensation at the heterointerface.^67^

Reactive site accessibility, catalyst dispersion, and dye interaction are all impacted by these colloidal characteristics.

### Adsorption behavior of RB5 on CZC

**Fig. 8:**
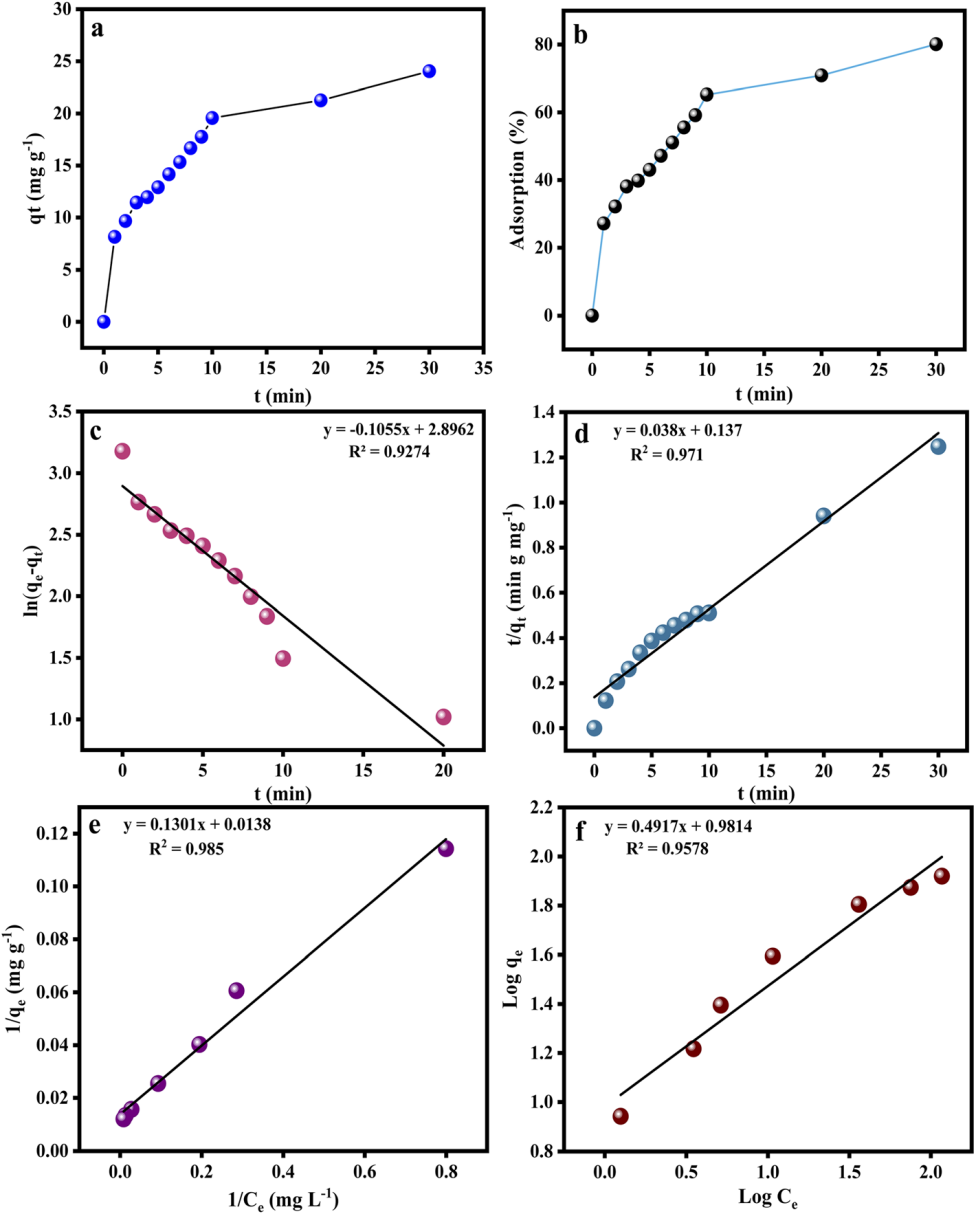
RB5 adsorption analysis on CZC: (a) adsorption capacity (*q*_*t*_), (b) adsorption percentage (%), (c) PFO and (d) PSO kinetic fits, and (e) Langmuir and (f) Freundlich isotherm fits.

Adsorption experiments were first conducted to clarify the dark removal contribution before interpreting the light-assisted process. The tests were performed using 30 mg L^−1^ RB5, 1 g L^−1^ catalyst, 50 mL solution volume, pH 7.32, and 30-min dark contact time.^65^ The adsorption capacity (*q*_*t*_) increased rapidly during the first 10 min and then entered a slower uptake regime, reaching approximately 24 mg g^−1^ after 30 min (Fig. 8a). The corresponding dark adsorption reached nearly 80% (Fig. 8b).^68^ This fast initial uptake can be attributed to abundant accessible surface sites, electrostatic interactions, and possible π–π interactions between CQD-derived carbon domains and the aromatic RB5 structure. The slower later-stage uptake reflects progressive occupation of remaining sites and diffusion limitations.^69^ According to the adsorption kinetic fits, the PSO model (*R*^2^ = 0.971) (Fig. 8c) provided a more accurate description of the data than the PFO model (*R*^2^ = 0.927) (Fig. 8d).

The Langmuir model (*R*^2^ = 0.985) (Fig. 8e) provided a slightly better fit than the Freundlich model (*R*^2^ = 0.958) (Fig. 8f), suggesting that adsorption occurs mainly through a finite set of active sites with a monolayer-like contribution, although surface heterogeneity remains evident from the reasonable Freundlich fit (Fig. 8e and f).^70^ The parameters derived from the linear fits are summarized in Table 6.

**Table 6:** Adsorption kinetic and isotherm parameters derived from the linear fits in Fig. 8

Model	Linear fit	Key parameter (s)	*R* ^2^
Pseudo-first order	ln(*q*_e_ − *q*_*t*_) = −0.1055*t* + 2.8962	*k*_1_ = 0.1055 min^−1^	0.9274
Pseudo-second order	t/*q*_*t*_ = 0.038*t* + 0.137	*q*_e_, cal ≈ 26.3 mg g^−1^	0.9710
Langmuir	1/*q*_e_ = 0.1301(1/*C*_e_) + 0.0138	*q*_m_ ≈ 72.5 mg g^−1^; *K*_L_ ≈ 0.106 L mg^−1^	0.9850
Freundlich	log *q*_e_ = 0.4917 log *C*_e_ + 0.9814	*n* ≈ 2.03; *K*_f_ ≈ 9.58	0.9578

### Contact time study

**Fig. 9:**
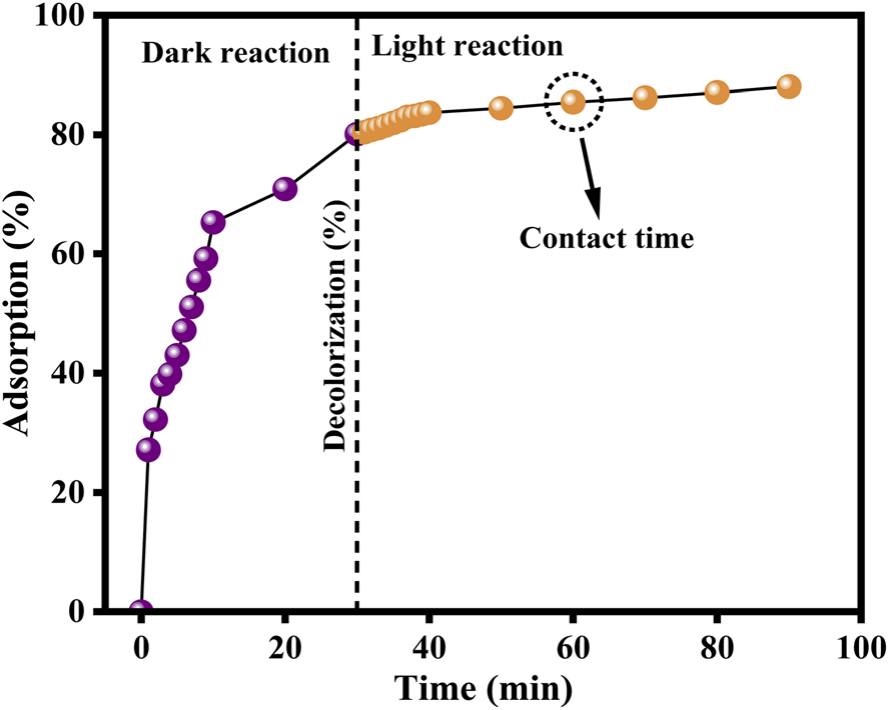
Effect of contact time on RB5 removal by CZC, showing dark adsorption followed by visible-light-assisted decolorization.

Dark reaction followed by light-assisted RB5 removal, as depicted in Fig. 9. The rapid dark stage produced approximately 80% removal by 30 min. Subsequent visible-light irradiation increased decolorization to almost 88% by 90 min. Results showed that CZC has an effective interaction with RB5 dye. The relatively small additional gain under illumination indicates that adsorption is a major contributor to color removal in this system. Therefore, the process is best described as adsorption-assisted photocatalytic decolorization rather than pure photocatalytic mineralization. This distinction is important for environmental interpretation because decolorization alone does not prove complete degradation or detoxification of RB5.

### Operating-parameter optimization

**Fig. 10:**
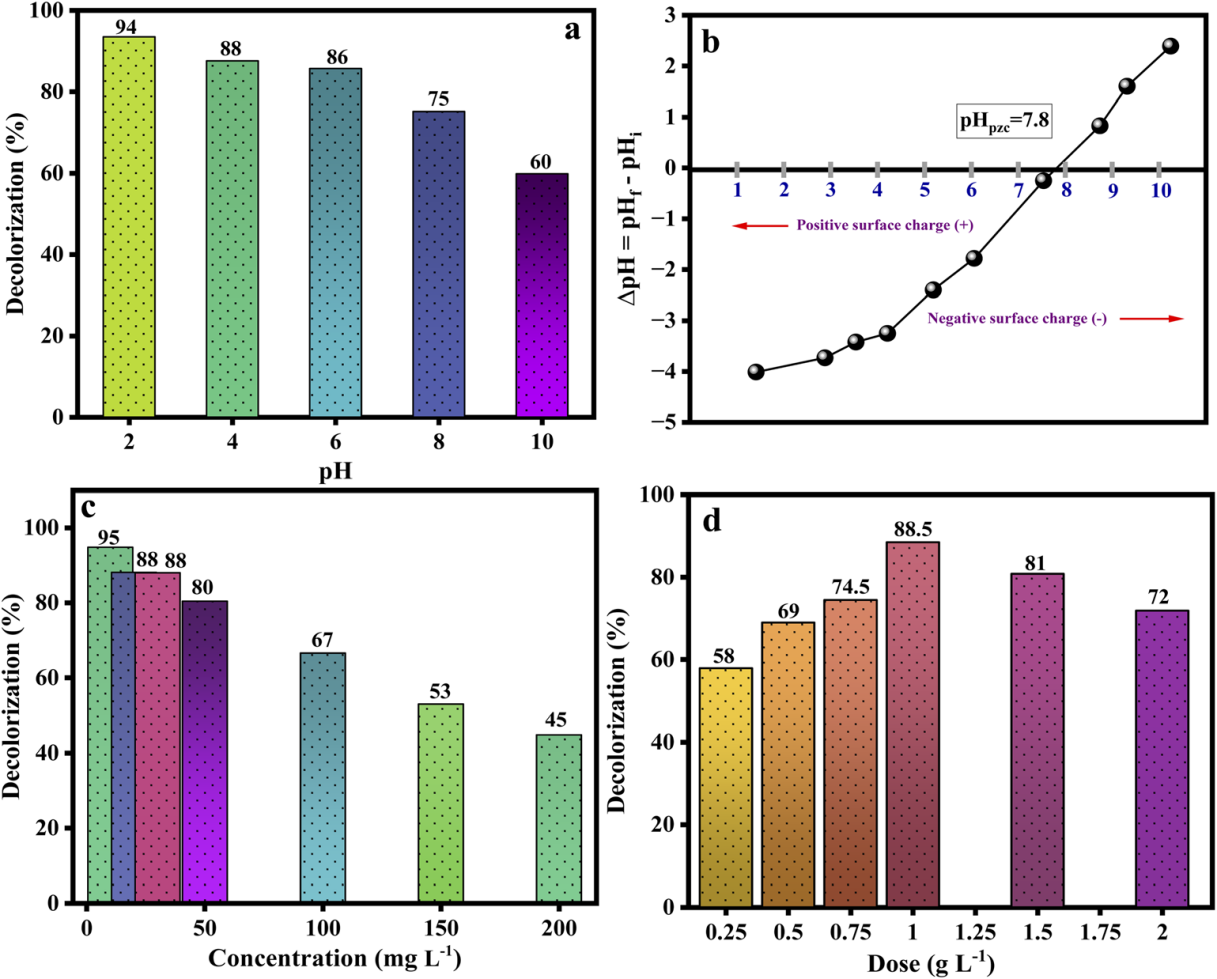
Effects of operating parameters on RB5 decolorization by CZC: (a) pH, (b) point of zero charge, (c) initial RB5 concentration, and (d) catalyst dose.

Fig. 10 summarizes the effects of pH, point of zero charge (pH_p_zc), initial dye concentration, and catalyst dose. Acidic conditions produced the highest RB5 decolorization, with 94% removal at pH 2, followed by 88% at pH 4, 86% at pH 6, 75% at pH 8, and 60% at pH 10 (Fig. 10a).^71^ The pH_p_zc of CZC was estimated to be 7.8 (Fig. 10b). Below this value, the catalyst surface is expected to be more positively charged, favoring adsorption of anionic RB5 species. Above pH_p_zc, the surface becomes more negatively charged, increasing electrostatic repulsion and lowering decolorization efficiency. This behavior confirms that surface charge and adsorption strongly influence the overall removal process. For initial RB5 concentrations, the decolorization decreased from 95% at 10 mg L^−1^ to 45% at 200 mg L^−1^ for high RB5 concentration (Fig. 10c). The ratio of reactive species to dye molecules drops, active sites become saturated, and dye screening reduces light penetration at higher dye concentrations. Catalyst loading study showed that 1 g L^−1^ was found to be a suitable amount, resulting in 88% decolorization (Fig. 10d).^72^

Higher doses reduced performance, perhaps due to particle aggregation and light-scattering effects, whereas lower doses offered inadequate active surface area. These findings suggest that for the best performance, both photonic efficiency and adsorption capacity must be matched.^73^

**Fig. 11:**
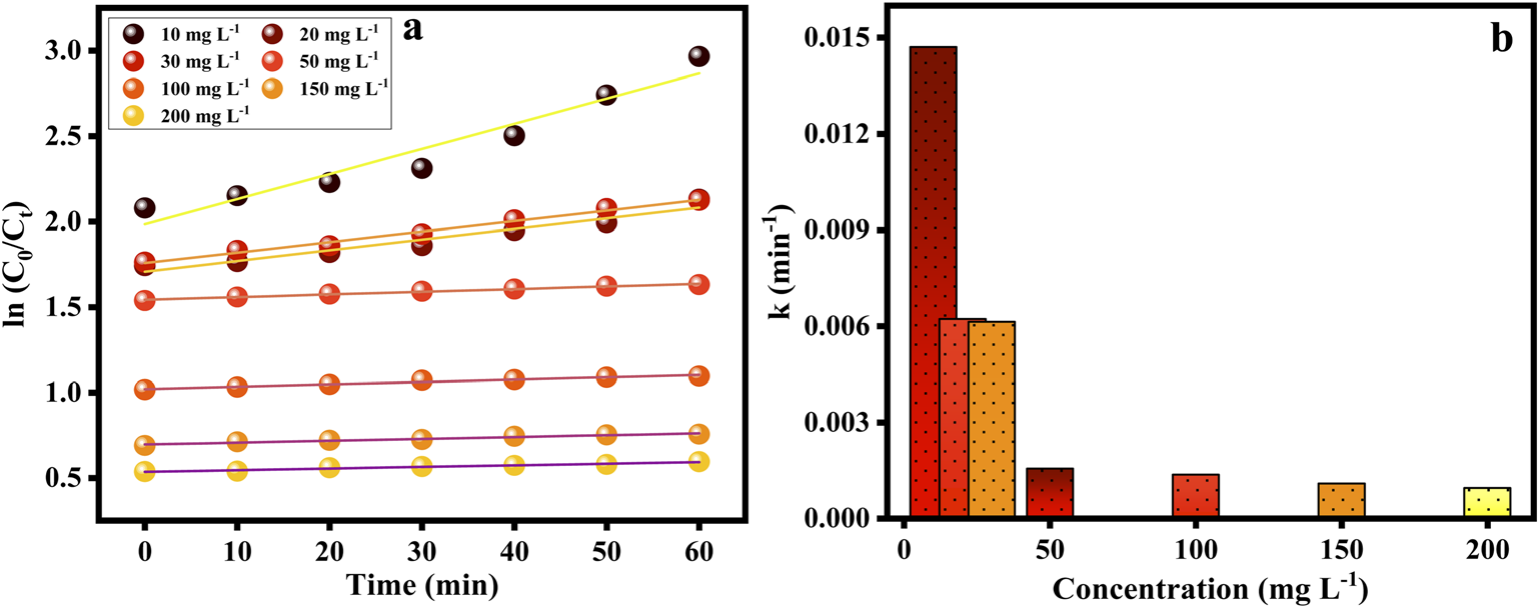
Pseudo-first-order kinetic analysis of RB5 decolorization using CZC at different initial RB5 concentrations: (a) ln(*C*_0_/*C*_*t*_) *versus* irradiation time (*t*) and (b) apparent rate constant as a function of initial RB5 concentration.

Fig. 11a displays a pseudo-first-order kinetic analysis supported by the linear relationship between ln(*C*_0_/*C*_*t*_) and irradiation time during the light-assisted process at different initial concentrations of RB5. The apparent rate (*k*_app_) gradually decreased as the initial dye concentration rose, which is in line with reduced photon penetration, active-site competition, and a lower reactive-species-to-dye ratio with higher dye loading (Fig. 11b).^38^ The kinetic constants should be interpreted as apparent decolorization descriptors under the selected conditions because adsorption and photocatalysis occur sequentially and partially overlap in the overall process.^38^

### Proposed charge-transfer mechanism and reactive pathways

**Fig. 12:**
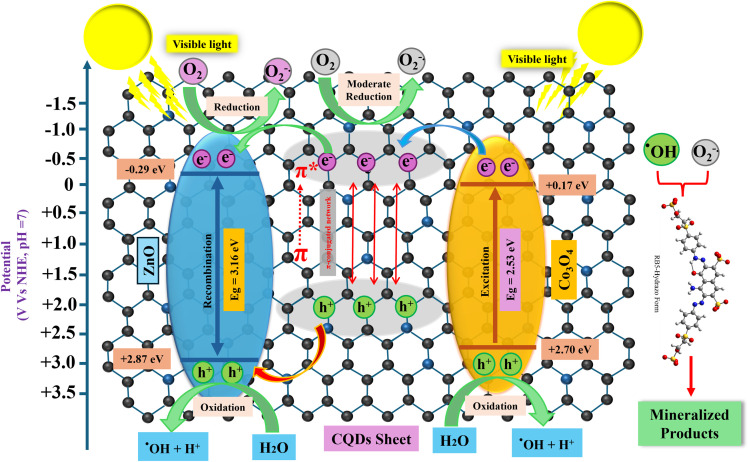
Proposed CQD-mediated Z-scheme charge-transfer mechanism in CZC for adsorption-assisted visible-light RB5 decolorization.

Fig. 12 presents the proposed CQD-mediated Z-scheme-like charge-transfer pathway for CZC. Under visible-light irradiation, Co_3_O_4_ and CQD-related domains can be photoactivated, while ZnO contributes strong redox potential when excited, which is responsible for oxygen radical generation, O_2_˙^−^, and attacks on pollutant molecules. Based on band-position analysis, lower-energy electrons in the Co_3_O_4_ conduction band may recombine with holes in the ZnO valence band through CQD-mediated interfacial pathways, leaving more reducing electrons on ZnO and more oxidizing holes on Co_3_O_4_/ZnO-related sites for reactive oxygen species formation.^74^ Hydroxyl radicals (^˙^OH) are primarily generated by holes in the valence band of ZnO, while Co_3_O_4_ contributes only weakly due to its less positive valence band potential. The interpretation is consistent with PL quenching, the narrowed effective band gap, and the improved visible-light response of CZC. The poor performance of Co-rich binary compositions such as 1ZnO : 3Co_3_O_4_ (28% decolorization) further suggests that excess Co_3_O_4_ may promote non-productive recombination or reduce the availability of strongly oxidizing ZnO-related holes.

In this proposed framework, CQDs serve as conductive bridges, photosensitizing domains, and adsorption-active surface components. Their functional groups promote RB5 adsorption and intimate contact with ZnO/Co_3_O_4_, while their carbonaceous structure facilitates interfacial charge migration. The mechanism remains a proposed model because direct radical detection and photoelectrochemical measurements were not performed; nevertheless, the combined UV-visible, PL, band-position, structural, and scavenger results provide consistent support for CQD-mediated charge separation and reactive-pathway enhancement.^75^

### Band edge calculations

The band-edge positions of ZnO and Co_3_O_4_ were estimated using the Mulliken electronegativity method.

Where *E*_CB_ is the conduction-band potential, *E*_VB_ is the valence-band potential, *X* is the absolute electronegativity of the semiconductor, *E*_e_ is the energy of free electrons on the hydrogen scale (approximately 4.5 eV), and *E*_g_ is the optical band gap (Table 7).

**Table 7:** Calculated band positions of ZnO and Co_3_O_4_ nanoparticles

Semiconductors	*E*_g_ (eV)	*E*_CB_ (eV)	*E*_VB_ (eV)
ZnO	3.16	−0.29	2.87
Co_3_O_4_	2.53	0.17	2.70

For ZnO nanoparticles, the band-edge positions are:*E*_g_ = 3.16 eV*X*_ZnO_ = 5.79 eV

Conduction-band position
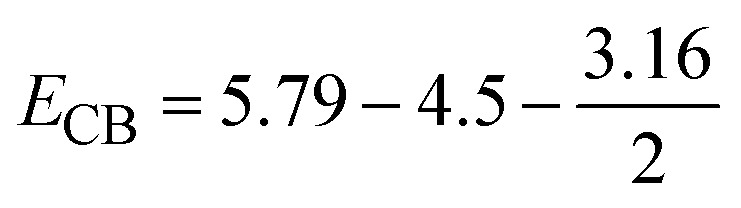

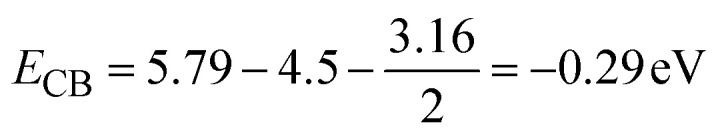


Valence-band position*E*_VB_ = −0.29 + 3.16*E*_VB_ = −0.29 + 3.16 = 2.87 eV

Thus, the calculated ZnO band positions are:

For Co_3_O_4_ nanoparticles, the band-edge positions are:

Given:*E*_g_ = 2.53 eV*X*_Co_3_O_4__ = 5.93 eV

Conduction-band position
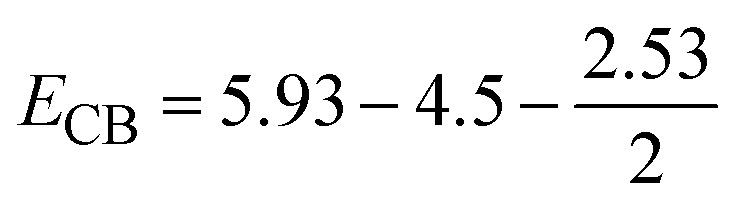

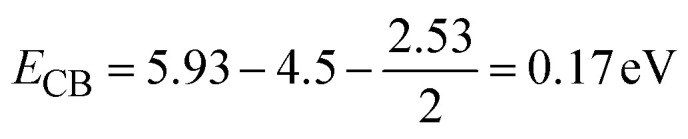


Valence-band position*E*_VB_ = 0.17 + 2.53*E*_VB_ = 0.17 + 2.53 = 2.70 eV

These potentials are reported *versus* the normal hydrogen electrode (NHE).

### Role of scavengers and recyclability

**Fig. 13:**
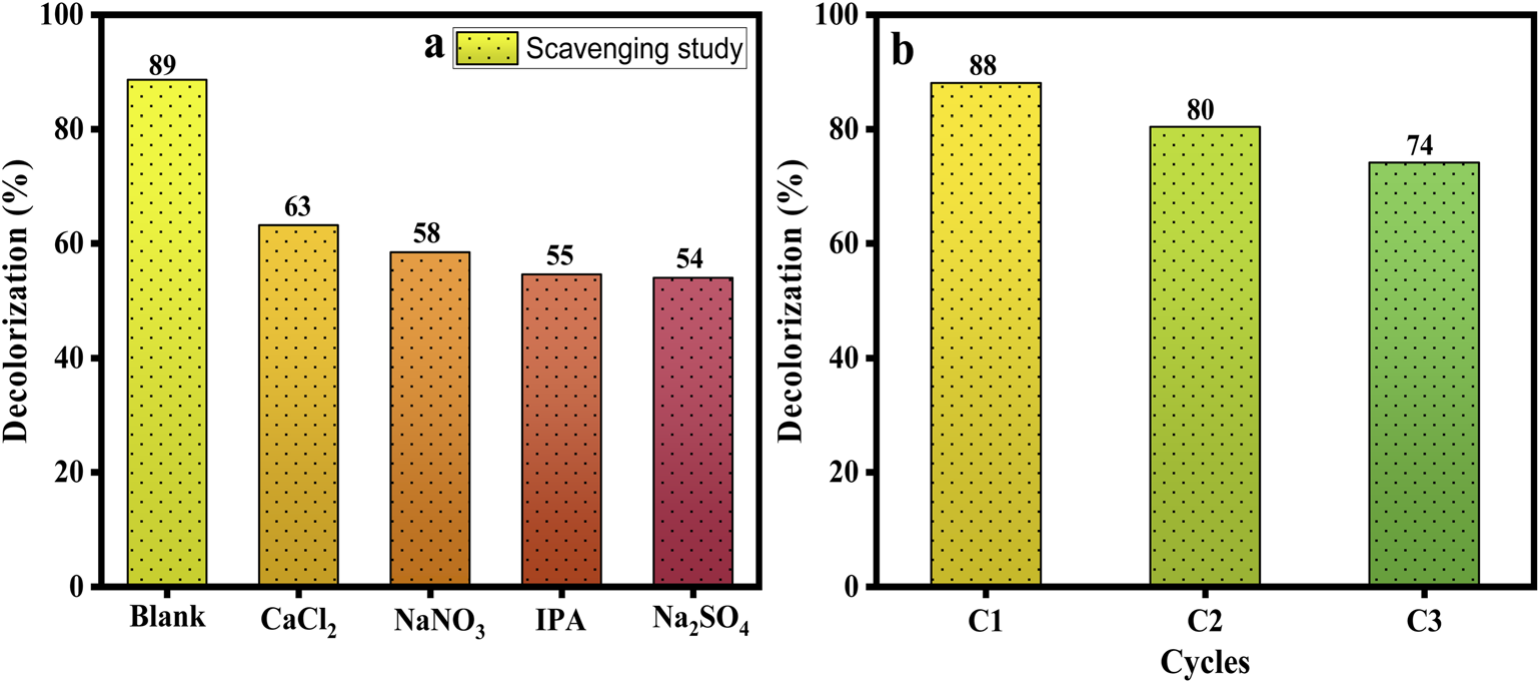
Mechanistic and stability tests: (a) scavenger effects on RB5 decolorization and (b) recyclability over three repeated cycles.

RB5 decolorization decreased from 89% in the blank test to 63% with NaNO_3_, 58% with CaCl_2_, 55% with IPA, and 54% with Na_2_SO_4_ in scavenger trials (Fig. 13a). The significant inhibition brought on by IPA and Na_2_SO_4_ indicates significant contributions from hole (h^+^) and hydroxyl radical (^˙^OH) associated pathways.^38,39^ Cl^−^ and NO_3_^−^ ions from NaNO_3_ and CaCl_2_ indicate suppression of photocatalytic degradation by competing for active sites and scavenging photogenerated reactive species, particularly h^+^ and ^˙^OH radicals, thereby reducing charge-transfer efficiency and pollutant oxidation rates.^76^ Therefore, a multi-pathway process comprising adsorption, direct hole oxidation, and hydroxyl radical generation is supported by the scavenger results.^77^ The stability of CZC was confirmed over the course of three cycles; CZC maintained 88%, 80%, and 74% decolorization, according to recyclability data (Fig. 13b). The drop in removal decolorization efficiency can be associated with surface fouling, catalyst loss during recovery, partial deactivation of active sites, and inadequate desorption of dye/intermediates. However, after three cycles, the catalyst maintained the majority of its activity, suggesting modest stability and reusability.^78^

### Machine-learning-assisted descriptor analysis

The Pearson correlation heatmap shows that removal efficiency is most strongly associated with the initial pollutant concentration. Higher initial concentrations significantly lowered removal efficiency, giving a strong negative correlation (*r* = −0.92). This trend agrees with the kinetic and optimization results, where higher dye loadings reduced photon penetration, increased active-site competition, and decreased the reactive-species-to-dye ratio. Compared with concentration, the remaining descriptors showed much weaker linear relationships with removal efficiency.^79^

pH exhibited a weak-to-moderate negative correlation (−0.25), consistent with improved RB5 removal under acidic conditions. Catalyst dose and irradiation time showed much weaker correlations, reflecting the fact that the tested dose range contained both beneficial surface-area effects and adverse light-scattering effects, while much of the removal occurred rapidly during the adsorption stage. Descriptor–descriptor correlations were generally weak, indicating that the feature-importance trends were not mainly caused by strong collinearity among the input variables.^80^

### Permutation feature-importance analysis

**Fig. 14:**
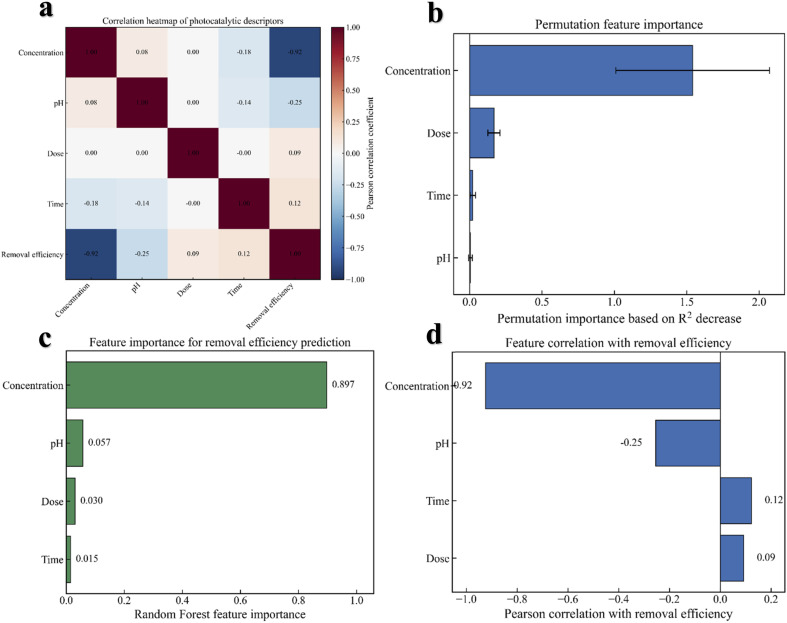
(a) Correlation heatmap of photocatalytic descriptors (b) permutation feature importance for removal efficiency prediction (c) random forest feature importance for removal efficiency prediction (d) Pearson correlation between individual descriptors and removal efficiency.

Permutation importance gave the same overall conclusion: initial RB5 concentration contained the most critical predictive information for removal efficiency. Shuffling concentration caused the largest decline in model performance, confirming that concentration dominates the experimental response within the tested parameter space. Dose showed a smaller positive contribution, whereas pH and time had limited additional predictive effect after concentration was accounted for (Fig. 14b).^81^

### Random forest feature-importance analysis

Random Forest feature importance provided a nonlinear model-based ranking of descriptor relevance. Nearly 90% of the model decision-making was associated with initial pollutant concentration, confirming that concentration was the dominant descriptor controlling CZC decolorization efficiency. The remaining variables contributed much less: pH ranked second with an importance score of 0.057, followed by dose (0.030) and time (0.015) (Fig. 14c). These low scores do not mean that pH, dose, and time are physically unimportant; rather, they indicate that their effects were relatively small compared with concentration within the available experimental dataset.^82^

### Individual feature correlation with removal efficiency

The target-correlation bar chart further confirms that concentration is the dominant linear descriptor. Its correlation coefficient (−0.92) is much larger in magnitude than those of pH, dose, and time, reinforcing the conclusion that dye loading controls the overall decolorization response. The negative pH correlation agrees with the surface-charge interpretation based on pH_p_zc, although its magnitude is much smaller than that of concentration.^83^

### Overall interpretation of descriptor analysis

Overall, both correlation and machine-learning analyses identify initial pollutant concentration as the primary factor governing CZC removal efficiency. The analysis supports the experimental interpretation that mass-transfer, light-screening, and active-site competition effects become increasingly important at higher RB5 concentrations. Because the dataset is small and derived from the present experimental design, the machine-learning results should be interpreted as an internal descriptor-ranking tool rather than a broadly generalizable predictive model.

Future studies should expand the dataset using factorial experimental designs and include additional descriptors such as light intensity, catalyst recovery loss, total organic carbon removal, and intermediate formation. Such data would allow the individual and interaction effects of pH, dose, contact time, and concentration to be separated more reliably and would strengthen process-level optimization.

### Catalytic significance and limitations

The present results show that fish-scale-derived CQDs improve the optical and interfacial properties of ZnO/Co_3_O_4_ and enable high RB5 color removal under mild conditions. At the same time, the data indicate that adsorption is a major contributor to performance, with approximately 80% removal occurring during the dark stage. Therefore, the catalytic significance of CZC lies in its integrated adsorption-assisted photocatalytic function rather than in photocatalysis alone. The mechanistic model is supported by optical, PL, band-position, structural, and scavenger evidence.

## Conclusion

Fish-scale-derived CQDs were successfully incorporated into a ZnO/Co_3_O_4_ heterostructure to form a ternary CZC nanocomposite for adsorption-assisted visible-light decolorization of RB5. Structural and spectroscopic analyses confirmed the coexistence of wurtzite ZnO, spinel Co_3_O_4_, and CQD-derived carbonaceous domains, while optical and PL measurements showed broadened visible-light absorption, an effective band gap of 2.62 eV, and suppressed radiative recombination. CZC achieved rapid dark adsorption of 65.2% (10 minutes) and 80.1% within 30 minutes, followed by light-assisted decolorization, reaching approximately 88% removal under optimized conditions and 94% at pH 2. Adsorption followed pseudo-second-order kinetics and was better described by the Langmuir model, with an estimated *q*_max_ of 72.5 mg g^−1^. Decolorization kinetics follow PFO kinetics, with a rate constant *K*_app_ decline with initial RB5 concentration. Scavenger and band-position analyses support a CQD-mediated Z-scheme-like pathway involving hole- and hydroxyl-radical-related routes. The catalyst retained 74% decolorization after three reuse cycles, indicating moderate recyclability. Exploratory descriptor analysis identified initial dye concentration as the dominant factor controlling removal efficiency. Overall, this work highlights fish-scale-derived CQDs as a sustainable strategy for enhancing ZnO/Co_3_O_4_-based adsorption-assisted photocatalytic dye removal, with CZC emerging as a promising photocatalyst for industrial wastewater treatment due to its efficient charge separation, visible-light activity, and stability.

## Author contributions

Javeria Asim: investigation, methodology, data curation, formal analysis, writing – review and editing Muhammad Younas Afzal: writing – original draft, writing – review and editing, visualization, methodology Saeed Rehman: investigation, formal analysis Ahson Jabbar Shaikh: validation, formal analysis, visualization Farooq Ahmad: investigation, validation, formal analysis Muhammad Tahir Amin: supervision, formal analysis, validation, investigation Fiaz Hussain: supervision, formal analysis, validation, visualization Muhammad Bilal: conceptualization, supervision, project administration, writing – review and editing, funding acquisition, validation, formal analysis.

## Conflicts of interest

The authors declare that they have no known competing financial interests or personal relationships that could have appeared to influence the work reported in this paper.

## Supplementary Material

RA-OLF-D6RA05073E-s001

## Data Availability

The datasets generated and analyzed during the current study, along with the machine learning code used in this work, are available from the corresponding author upon reasonable request. Supplementary information (SI): the synthesis procedures for fish-scale-derived CQDs, ZnO, Co_3_O_4_, and the CQDs/ZnO-Co_3_O_4_ nanocomposite. See DOI: https://doi.org/10.1039/d6ra05073e.
