# Supplementary material for: Fish-scale-derived carbon quantum dots coupled with ZnO/Co3O4 for adsorption-assisted visible-light decolorization of reactive black 5
Source: RSC Adv. 2026 Aug 18;16(41):48850–68. doi: 10.1039/d6ra05073e (PMC13485270; doi:10.1039/d6ra05073e)
Supplement: Supplementary file 1 [file RA-016-D6RA05073E-s001.pdf]

Supplementary Information (SI) for RSC Advances.
This journal is © The Royal Society of Chemistry 2026

Fish-Scale-Derived Carbon Quantum Dots Coupled with ZnO/Co_3O_4 for Adsorption-Assisted Visible-Light Decolorization of Reactive Black 5

Javeria Asim[a,†], Muhammad Younas Afzal[a,b,†], Saeed Rehman[c], Ahson Jabbar Shaikh[b], Farooq Ahmad[d], Muhammad Tahir Amin[e, *], Fiaz Hussain[f, *], Muhammad Bilal[a, *]

[a]Department of Environmental Sciences, COMSATS University Islamabad, Abbottabad Campus, Abbottabad - 22060, KPK, Pakistan

[b]Department of Chemistry, COMSATS University Islamabad, Abbottabad Campus, Abbottabad - 22060, KPK, Pakistan

[c]School of Environment and Resources, Laboratory of Solid Waste Treatment and Resource Recycle, Southwest University of Science and Technology, Mianyang, 621010, China

[d]Department of Chemical and Materials Engineering, College of Engineering, Northern Border University, Arar, 91431, Saudi Arabia

[e]Civil and Environmental Engineering Department, College of Engineering, King Faisal University, Al-Ahsa 31982, Saudi Arabia, mqdir@kfu.edu.sa

[f]Department of Mechanical Engineering, Gachon University, Seongnam-si, Gyeonggi-do, 13120, South Korea, fiaz415@gachon.ac.kr

† These authors contributed equally to this work.

Corresponding authors:

*mbilal@cuiatd.edu.pk (Prof. Dr. Muhammad Bilal)

*mqdir@kfu.edu.sa (Muhammad Tahir Amin)

*fiaz415@gachon.ac.kr (Fiaz Hussain)

Supplementary Information

Synthesis Procedure for Fish-Scale-Derived CQDs, ZnO, Co_3O_4, and CQDs/ZnO-Co_3O_4

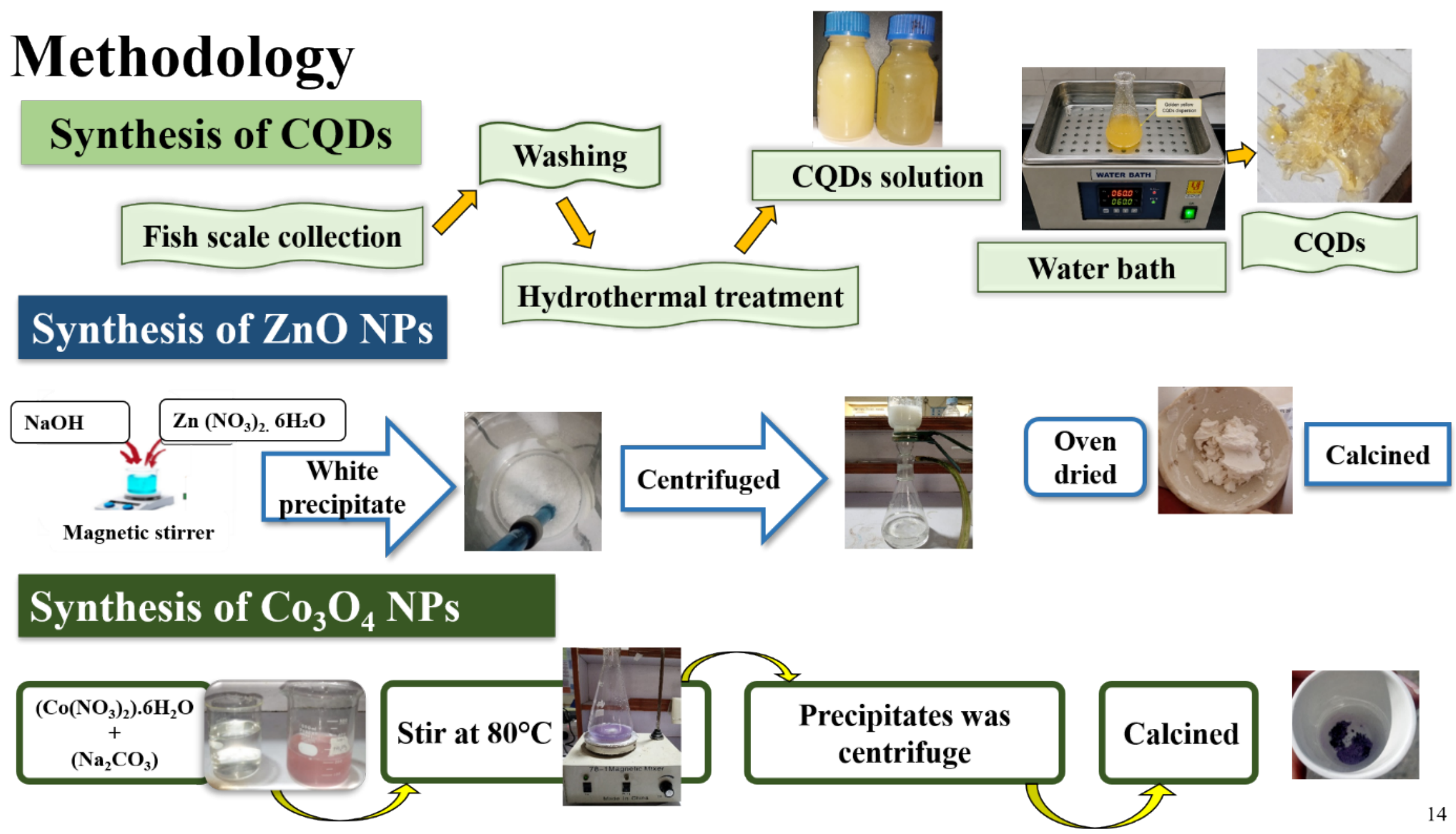

Nanocomposite

Figure S1. Synthesis of CQDs, ZnO, and Co_3O_4 nanoparticles.

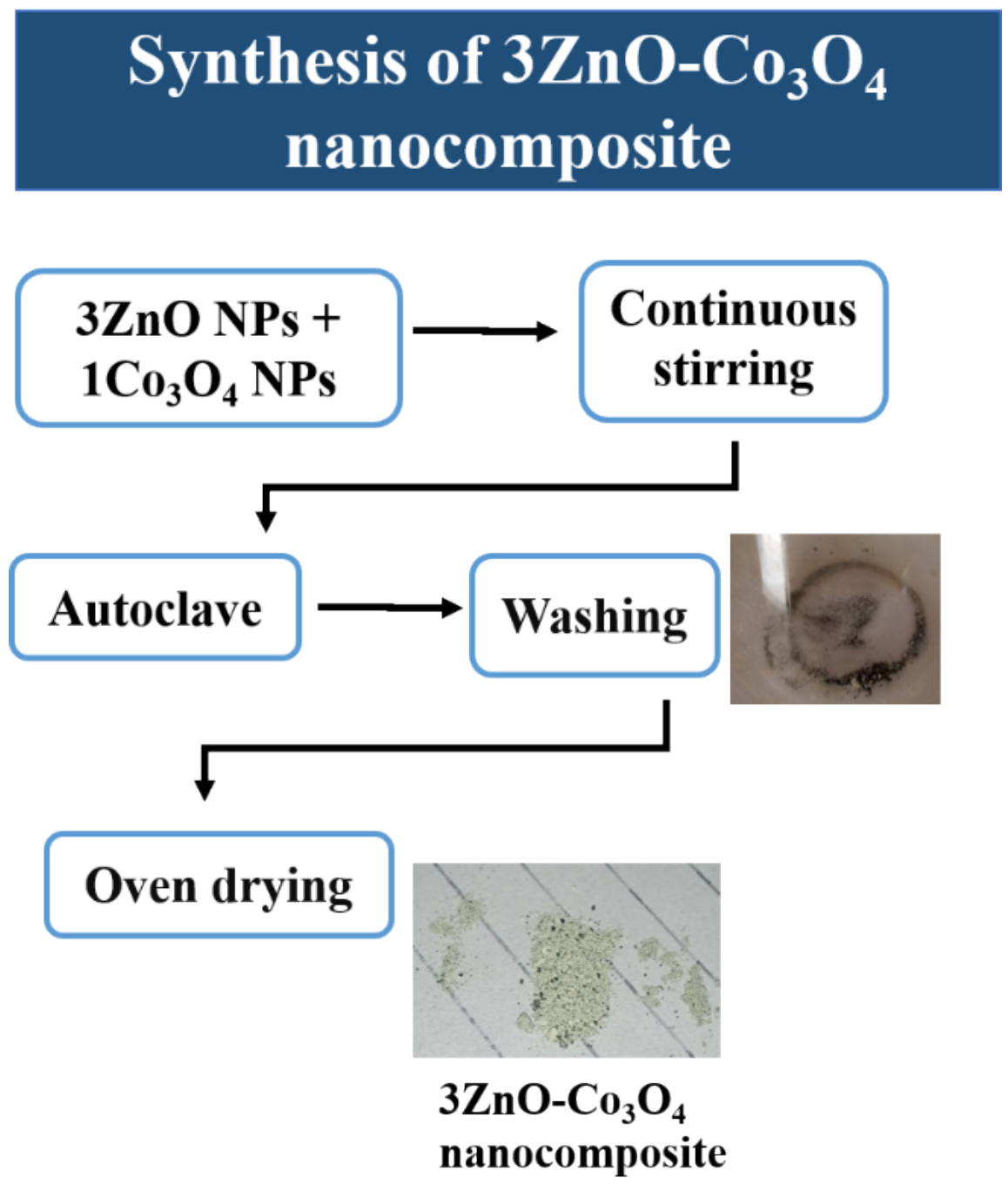

Synthesis of 3ZnO-Co_3O_4

Figure S2. Synthesis of 3ZnO/Co_3O_4 binary nanocomposites.

Synthesis of 0.5CQD/3ZnO-Co_3O_4

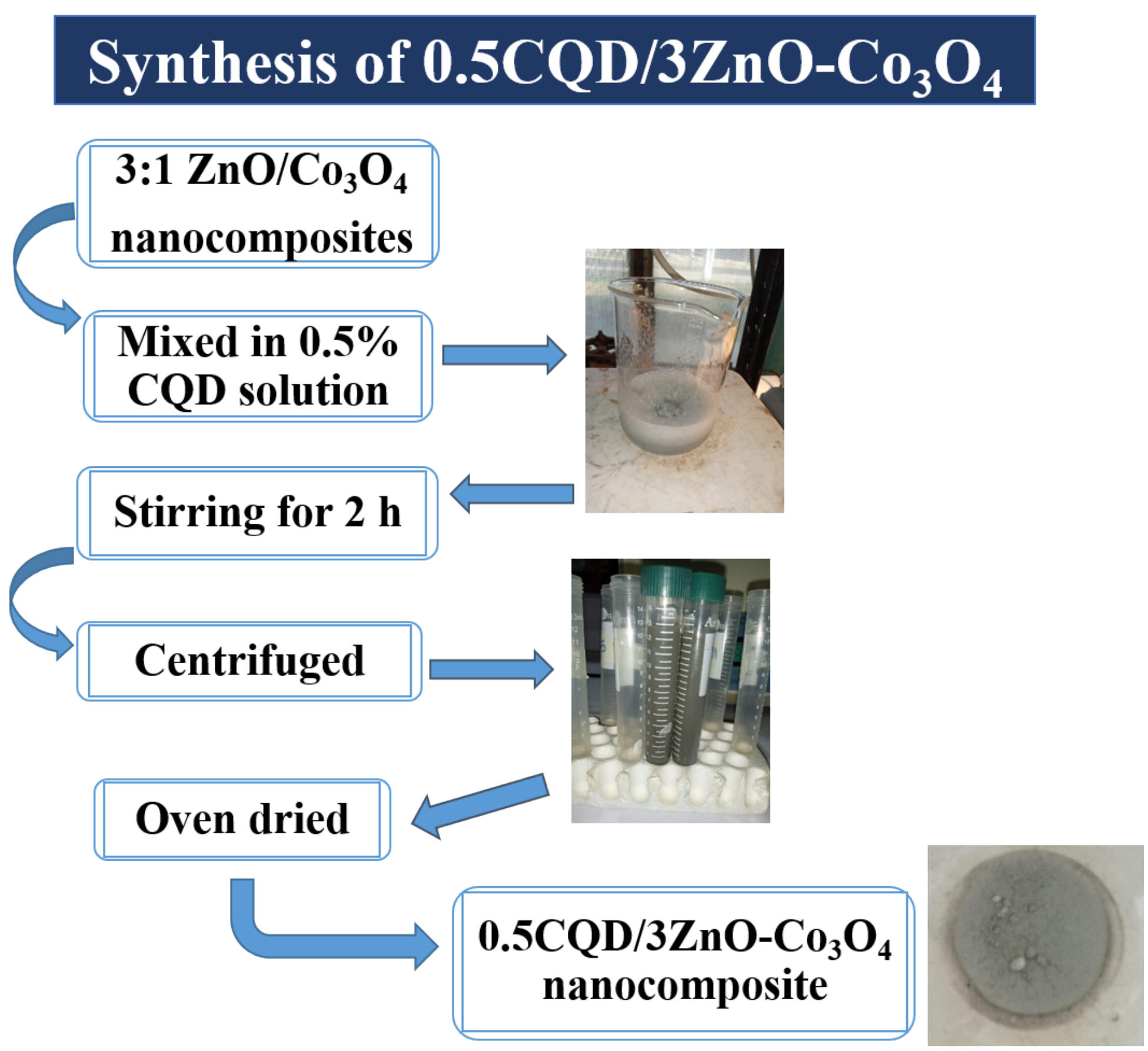

Figure S3. Synthesis of 0.5CQD/ZnO-Co_3O_4 ternary nanocomposites.
